# Coherent controlization using superconducting qubits

**DOI:** 10.1038/srep18036

**Published:** 2015-12-15

**Authors:** Nicolai Friis, Alexey A. Melnikov, Gerhard Kirchmair, Hans J. Briegel

**Affiliations:** 1 Institute for Theoretical Physics, University of Innsbruck, Technikerstraße 21a, A-6020 Innsbruck, Austria; 2 Institute for Quantum Optics and Quantum Information, Austrian Academy of Sciences, Technikerstraße 21a, A-6020 Innsbruck, Austria; 3 Institute for Experimental Physics, University of Innsbruck, Technikerstraße 25, A-6020 Innsbruck, Austria

## Abstract

Coherent controlization, i.e., coherent conditioning of arbitrary single- or multi-qubit operations on the state of one or more control qubits, is an important ingredient for the flexible implementation of many algorithms in quantum computation. This is of particular significance when certain subroutines are changing over time or when they are frequently modified, such as in decision-making algorithms for learning agents. We propose a scheme to realize coherent controlization for any number of superconducting qubits coupled to a microwave resonator. For two and three qubits, we present an explicit construction that is of high relevance for quantum learning agents. We demonstrate the feasibility of our proposal, taking into account loss, dephasing, and the cavity self-Kerr effect.

The ability to coherently control and manipulate individual quantum systems lies at the heart of modern quantum technologies and applications in quantum information^1^,^2^,^3^. Any quantum computation can be realized as a sequence of elementary quantum gates^4^, which are highly-controlled quantum interactions of few qubits at a time, and quantum measurements. Prominent applications include, e.g., quantum algorithms for efficient factoring^5^,^6^ and quantum simulation^7^,^8^,^9^,^10^,^11^. More recently, applications of quantum algorithms to certain problems in machine learning, including data classification^12^ and search engine ranking^13^,^14^, have been proposed. Other recent proposals, which are of particular interest for the current paper, are the quantum-enhanced deliberation of learning agents in the context of quantum artificial intelligence^15^,^16^ and the notion of autonomous and adaptive devices for quantum information processing^17^. In parallel to these theoretical developments, the design of experimental implementations of quantum computational architectures in systems such as, e.g., trapped ions^18^,^19^,^20^,^21^ and optical setups^22^,^23^,^24^ has been greatly advanced.

In addition, the progress in controlling superconducting (SC) quantum systems^25^,^26^,^27^,^28^ has significantly strengthened the role of SC qubits (see, e.g.,^29^,^30^) as contenders for the realization of quantum computational devices. In particular, 1-D^31^ and 3-D^32^,^33^ transmons — SC qubits impervious to charge noise — appear as promising candidates. Initial studies of complex algorithms and gate operations^34^,^35^, fault-tolerance^36^,^37^,^38^, and hardware efficiency^39^ in such systems further raise the hopes for a scalable quantum computational architecture using SC circuitry. Moreover, coupling SC qubits via microwave resonators permits access to the domain of cavity QED^40^,^41^,^42^. There, the exceeding level of control over the combined system can be utilized, e.g., to resolve photon number states^43^, or to deterministically encode quantum information in the resonator states^44^,^45^. We shall draw from this rich quantum optics tool-box in the following.

Notwithstanding these developments, a paradigm with respect to which all of the above implementations are typically deemed successful is the realization of the unitaries of a universal set of quantum gates, see, e.g., Refs 3,46. While a universal set of gates in principle allows any unitary to be efficiently approximated, there are some tasks for which this approach lacks a certain *flexibility*. For instance, when specific subroutines of an algorithm require modifications in between individual runs. Prominent examples include the period-finding subroutine in Shor’s algorithm^6^, which is typically used several times for different functions throughout the algorithm, or when the phases corresponding to different unitaries are to be estimated using Kitaev’s scheme^47^. This issue is also of particular importance when quantum subroutines are used in the decision-making of learning agents^48^, which update their subroutines based on experience gathered throughout the learning process. In all these cases, a set of unitaries, which are applied conditionally on the states of an ensemble of auxiliary qubits, is modified in subsequent applications of the subroutines. It is hence of significant interest to establish a method of *coherent controlization* — a mapping from a set of unitaries on a target Hilbert space to a single controlled operation on a larger (control & target) space — that is independent of the chosen set of unitaries.

Unfortunately, it is not possible to design generic quantum circuits that achieve this conditioning independently of the selected unitaries when only single uses of the unitaries in question are permitted^49^,^50^,^51^. On the other hand, physical implementations of the unitaries that are to be controlled are typically already conditioned on fixing some degrees of freedom such as spatial locations (a laser beam illuminating an ion; an optical element being placed in the path of a light beam), or resonance frequencies. This practically allows the realization of purpose-built schemes that “add control” to unspecified unitaries, e.g., in optical setups^52^, or trapped ions^48^,^51^.

Here, we propose a *modular* and *adaptive* implementation of coherent controlization in a superconducting system of transmon^31^ qubits coupled to a microwave resonator. In the dispersive limit, the coupling between these systems can be understood as well-resolved shifts of the cavity frequencies, dependent on the qubit states, or vice versa, shifts of the qubit frequencies conditioned on the cavity state. Based on this principle, our protocol is assembled from unconditional displacements of the cavity mode and qubit operations conditioned on the vacuum state of the resonator, similar as in Refs 44,45. We present a detailed construction of our protocol for two and three qubits, and we give a recipe for up-scaling our scheme to an arbitrary number of qubits. As a cornerstone of our investigation, we include an in-depth analysis of effects detrimental to the success of our protocol. For the strongest source of errors, the cavity-self Kerr, we provide analytical estimates of the disturbance, and discuss methods to reduce it. Using numerical simulations to further take into account decoherence effects such as amplitude- and phase-damping, we hence show that our scheme for coherent controlization can be implemented using current superconducting technology.

## Results

The remainder of this paper is organized as follows. We first briefly review the basic concepts for our proposal: We give a definition for coherent controlization and illustrate it by an application to learning agents in quantum artificial intelligence, before we give a short description of the superconducting transmon qubits that we consider here. We then introduce the conceptual centrepiece, the coherent controlization protocol for two superconducting qubits, where we first discuss the idealized basic protocol, before turning our attention to the influence of the Kerr effect and decoherence. Finally, we extend our protocol to three qubits and beyond.

### Framework

#### Coherently controlling unknown unitaries

At the heart of many quantum computational algorithms lie subroutines in which operations of choice on a finite-size register of qubits are performed conditionally on the state of a control qubit. This is the case, for instance, in Kitaev’s phase estimation subroutine^47^, where, the 2*^n^*^−1^-fold application 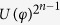 of a phase rotation 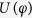 is executed only if the *n*-th ancilla qubit is in the state 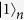. Similar subroutines feature also in Shor’s factoring algorithm^6^. When 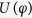 is specified, the corresponding subroutine can be efficiently approximated by combining operations from a set of universal quantum gates (see, e.g.,^46^). The decomposition into the universal gates, and their assembly to form the subroutine clearly requires some (classical) computational effort along with (some) knowledge of  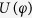. This becomes a practical impediment when a device implementing said subroutine is to be used consecutively for different choices of  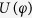, possibly diminishing any computational speed-up with respect to purely classical devices. In particular, this issue is of crucial interest for the design of quantum-enhanced autonomous learning agents^15^, where the quantum speed-up concerns the deliberation-time, and the agents need to update their subroutines throughout the learning process.

It would hence be highly desirable to have access to fixed global operations, let us call them *A* and *B*, on the target and control registers which allow turning an unspecified local operation 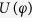 into its controlled version 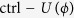, such that 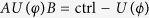. However, this requirement cannot be met by any fixed *A*, *B* for all 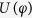, see^50^, and therefore, in particular, generic system independent controlization is not possible when the action of  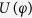 on the target Hilbert space is unknown. Fortunately, most practical realizations of (unitary) operations are not strictly local with respect to the target Hilbert space, but are already conditioned on some additional degrees of freedom. For instance, for quantum information encoded in photons, the optical elements must be placed in the beam path, conditioning the operations on spatial degrees of freedom. Another example are laser pulses driving transitions between qubit states encoded in ions, which must be at resonance, conditioning the transformations on the correct frequency. Such implicit conditioning on additional degrees of freedom can be exploited to “add control” to unitaries that are unknown in the sense specified above^51^,^52^.

Following Ref. 48, we shall refer to mappings from a set of operations 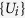 on the target Hilbert space 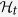 to operations *U* on the joint Hilbert space 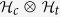 of the control and target systems, such that


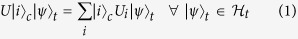


for some (orthonormal) basis 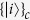 of 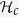, as well as to any specific physical realization of such mappings as *coherent controlization*. In the following, we shall discuss how general coherent controlization can be implemented in superconducting qubits, providing a modular and adaptive architecture for quantum computational tasks. For a detailed analysis of the *feasibility* and *scalability* of our proposal, we provide a concrete example for an application of coherent controlization in the decision process of learning agents, where the adaptive character of our proposal is of particular relevance.

#### Coherent controlization in the context of learning agents

In the model of projective simulation (PS)^16^, an autonomous learning agent draws upon previous experience to simulate its future situation in a given (and partially unknown) environment. The centrepiece of such an agent, which is also equipped with sensors (to receive perceptual input, *percepts*, from the environment) and actuators (enabling it to act on and change the environment) is a specific type of memory (ECM)^16^. In abstract terms, the memory is represented by a space of clips that can represent percepts, actions, and combinations thereof. After receiving sensory input, the PS agent initiates a random walk within the clip space to find an action. Under a given reward scheme, the agent’s choices have consequences that modify and update its memory, it *learns*. Throughout the learning process, the agent must hence be able to adjust its deliberation according to its experience, which entails updating the stochastic matrix 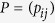 of transition probabilities of the random walk in the space of memory clips. Recently, the PS model has been shown to perform competitively in typical artificial intelligence benchmark tasks^53^, and, further, that its memory structure provides a dynamic framework for generalization^54^.

A particular variant of the PS that we shall focus on now, is reflecting projective simulation (RPS)^15^. In the RPS framework, the random walk in the memory space is continued until the underlying Markov chain 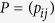 is (nearly) mixed, and actions are then sampled according to the stationary distribution *π*, where 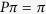. The quantized version of the RPS yields a quadratic speed-up with respect to its classical counterpart, both in the number of calls to *P* needed to mix the chain, and in the number of samples until an action is obtained. The Szegedy-type quantum random walk involved in this procedure requires several levels of coherent controlization. At the lowest level of this nested scheme of adding control, one encounters a set of unitaries 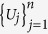 that encode the 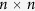 stochastic matrix *P* of an *n*-clip network. That is, the first column of 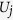 has real and positive entries 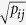 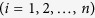, and each such *probability unitary* 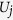 may thus be parameterized by 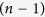 real angles 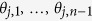. After each step of the learning process, the matrix *P* is updated according to the rewards that may have been incurred, requiring also updates of the 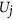. In the spirit of adaptiveness of the agent’s design it is desirable that these updates can be carried out by directly updating the angles 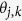 in an otherwise fixed hardware. A method^48^ for realizing this requirement is a nested construction of coherent controlization for 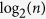 qubits, where control is added to 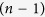 single-qubit *Y*-rotations 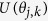 for each 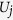.

**Figure 1:**
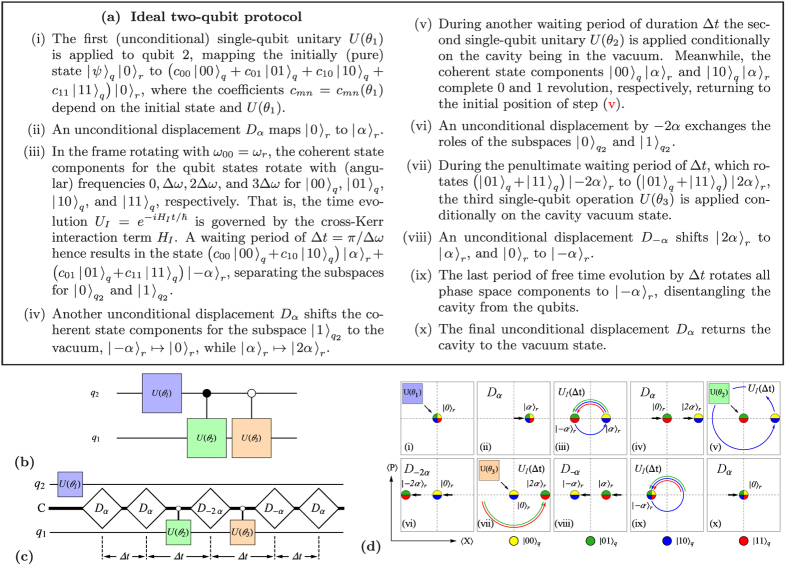
Two-qubit protocol for coherent controlization. (**a**) The steps (i)–(x) realize the probability unitaries 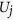 (positive, real entries in the first column), represented by the circuit diagram in (**b**). The filled dots “•” on the controlled operations indicate that the unitaries on the target are conditioned on the control qubit state 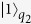, while the hollow dots “◦” denote conditioning on the control qubit state 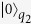, by way of coherent controlization of the three single qubit unitaries 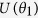, 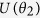, and 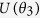. In (**c**), an extended circuit diagram is shown, where the upper and lower lines indicate qubits 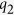 and 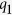, respectively, while the middle line labelled C represents the cavity mode. The white rectangles represent unconditional displacements, and the control lines between the cavity and the single-qubit operations indicate operations conditioned on the resonator being in the ground state. The waiting time between the displacements is 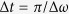. (**d**) shows the phase space representation of the protocol for the coherent state components corresponding to different qubit states.

For instance, for two-qubits, the unitary 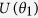 is unconditionally applied on the control qubit, followed by the applications of 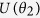 and 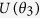 conditioned on the control qubit being in the states 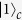 or 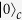, respectively, see Fig. 1(b). For three qubits, a pair of two-qubit subroutines of the form just described replaces the two conditional single-qubit operations, see Fig. 3(a), and so forth. This construction already entails the conditioning of single-qubit operations on all subspaces of the control qubits. We shall therefore consider the implementation of the probability unitaries 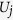 in superconducting qubits as a representative example that demonstrates the feasibility of coherent controlization. In order to proceed, we shall next give a brief overview of the properties of the superconducting qubit systems suitable for our purposes.

#### Superconducting qubits coupled to microwave resonators

The physical system that we consider in our proposal is an array of superconducting transmon qubits^31^ coupled to a microwave resonator. For the reader unfamiliar with the principal design of such a system, let us give an intuitive example. Consider a basic superconducting *LC*– circuit, which may be thought of as the realization of a quantum mechanical harmonic oscillator, where charge and flux take the role of the canonically conjugate variables. Via the non-linearity of Josephson-junctions, an anharmonicity can be introduced into the system, which modifies the otherwise equal energy level spacing. This allows one to frequency-address transitions between two chosen levels (typically the two lowest-lying levels), thus forming a qubit. For the practical realization of such a macroscopic qubit, several options, such as charge and flux qubits, are available and we direct the reader to pedagogic reviews (see, e.g., Ref. 29) for more details on their differences.

Here, we shall focus on the *transmon qubit*, introduced in Ref. 31, which is in its design similar to usual charge qubits, in the sense that two superconducting islands are connected via a Josephson junction with associated Josephson energy 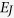. In some setups two Josephson junctions can be used to form a dc-SQUID. This leaves the possibility to modify the Josephson energy 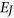 of the junctions by threading the SQUID with an external magnetic flux. Besides 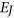, the energy levels of such charge qubits are determined by the charging energy 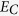 of the superconducting island. The departure of the transmon from other designs lies in the introduction of a large shunting capacitance parallel to the dc-SQUID, which drastically reduces 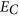. The transmon qubit is hence operated in a regime where 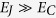, which leads to an exponential decrease of the charge dispersion in 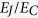, while the anharmonicity is only diminished polynomially in this ratio. In other words, the qubit levels remain addressable by frequency selection, while their sensitivity to environmentally induced charge noise is practically removed, which significantly improves qubit coherence times 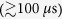
32,33.

For our investigation, several such transmon qubits shall be considered to be capacitively coupled to a superconducting resonator. To good approximation (see, e.g., the Supplementary Information), the Hamiltonian for this dispersive interaction may be written as^55^,^56^


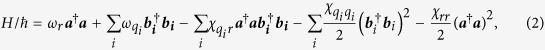


where 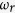 and 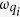 are the (angular) frequencies of the resonator mode and the *i*-th qubit, respectively, with the corresponding ladder operators 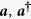, ***b***_***i***_, 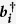. For the remainder of this paper, higher excitation numbers are ignored for the qubit-modes. Due to the coupling of the qubits to the resonator, the latter also acquires an anharmonicity, which causes the undesired self-Kerr effect^57^ represented by 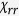. The constants 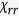, 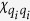, and the cross-Kerr coefficient 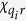 are hence not independent. For instance, when only one qubit is present, 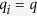, the relation between these parameters^55^ is 
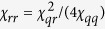
 in the dispersive limit. Typical values for these parameters that we will consider in the two-qubit case are 
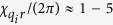
 MHz, and 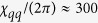 MHz (where we have assumed 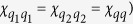, corresponding to cavity anharmonicities 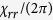 roughly between 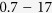 kHz.

The terms proportional to 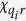 can be interpreted as conditional frequency shifts: Depending on the number of excited qubits, the resonator frequency 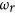 can be regarded as shifted by the sum of the corresponding values 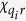. Conversely, the frequency of the *i*-th qubit can be considered as being shifted by the product of 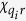 and the number of photons in the cavity. In the strong dispersive regime^45^, the spectral lines of the cavity for different qubit states are well-resolved, and, *vice versa*, so are the qubit transition frequencies for different numbers of excitations of the resonator. Both of these points of view will play an important role in our protocol for coherent controlization that we will introduce next.

### Coherent controlization for two superconducting qubits

In the protocol for coherent controlization that we propose here, the phase space of the resonator mode serves as a bus between the qubits. It enables the conditioning of operations on particular subspaces of the qubit Hilbert space by separating the resonator states corresponding to different qubit subspaces in phase space. The mechanism for this separation is the free time evolution of coherent states with different frequencies. Recall that the resonance frequency of the cavity depends on the state of the qubits. Similar to the procedures used in^44^,^45^,^58^, the following operations are employed for our protocol:

- **Unconditional displacements** *D*_*α*_: A very short pulse (a few ns), that is, sufficiently broad in frequency (of width 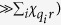, so as not to distinguish between the state-dependent frequencies of the resonator, displaces the cavity state independently of the state of the qubits.
- **Free time evolution** 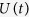: During the free time evolution of the cavity mode, governed by the corresponding parts of the Hamiltonian in (2), coherent states of the resonator corresponding to different qubit states rotate in phase space at different speeds. Appropriate waiting periods can hence be used to separate or recombine different coherent states.
- **Conditional qubit operations:** When the (average) photon number in the resonator is 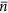, the (mean) qubit frequencies 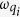 are shifted to 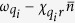 with a spread 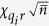. Addressing the qubits with signals sufficiently narrow in frequency around 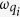 therefore conditions the single-qubit operations on the cavity vacuum state 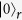. Unconditional qubit operations can be realized by appropriately broad (or multi-frequency) pulses.

With these operations available we shall now specialize to the case of two qubits.

#### Ideal two-qubit protocol

Let us first analyze an idealized situation where the cavity Kerr effect due to 
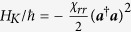
 and any loss due to interactions with the environment can be disregarded. Once we have set up our protocol for two qubits in this idealized setting, we shall consider the robustness of the protocol under the influence of the mentioned harmful effects. Irrespective of this restriction, the cross-Kerr interaction term 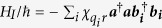 (in the strong dispersive regime) splits the cavity resonance frequency into 4 well-resolved spectral lines 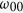, 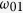, 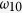, and 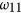, corresponding to the two-qubit basis states 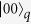, 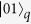, 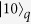, and 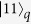. By selecting the qubit-cavity cross-Kerr coefficients 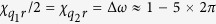 MHz, the spectral lines are equally spaced, i.e.,


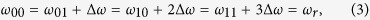


which can be abbreviated to 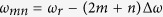. With the resonator prepared in the vacuum state 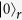, we now construct a protocol that implements coherent controlization for a set of three single-qubit unitaries 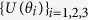 to realize the probability unitary


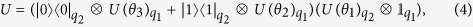


represented by the circuit-diagram in Fig. 1(b). The protocol, whose steps are detailed in Fig. 1(a) and which are illustrated in Fig. 1(b,c), can be decomposed entirely into the operations described above.

Given that the initial (unconditional) qubit rotation 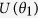 can be performed very rapidly (≈10 ns), the overall gate time 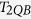 is approximately 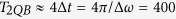 ns to 2 *μ*s [for 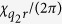 between 5 MHz and 1 MHz], compared to typical^44^ cavity coherence times *τ*_*r*_ ≈100 *μ*s, and qubit relaxation- and dephasing times of around 20–100 *μ*s. To maintain a high fidelity, especially when the protocol is extended to several qubits (as we shall do in the following), it is desirable to decrease the overall gate time, and hence to increase the qubit-cavity cross-Kerr coefficients 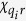. However, this can only be done at the expense of an increased cavity self-Kerr term proportional to 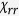, which grows quadratically with the 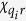. In other words, there is a trade-off between decoherence and imperfections of the gates due to distortions of the coherent-state components (through the Kerr effect). Before we extend our protocol to larger numbers of qubits, we will therefore study the robustness of the two-qubit protocol under these effects.

#### Influence of the cavity self-Kerr effect

With increasing coupling between the qubits and the resonator, the influence of the anharmonicities of the transmon qubits on the resonator mode becomes ever stronger. The ensuing cavity Kerr effect distorts (and displaces) the shape of the phase space distributions^57^. To estimate the impact on the performance of our protocol, we shall more closely inspect the three stages at which the overlap of the phase space distributions with the target states determines the success of the protocol. First, let us denote the overall state after step *k* as


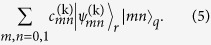


During steps (iv) and (vi), the coherent state components 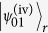, 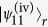 and 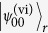, 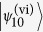, corresponding to the subspaces 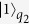 and 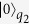, respectively, are required to significantly overlap with the vacuum to achieve the conditioning of 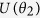 and 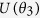. Finally, after being disentangled from the qubits in step (ix), all resonator states 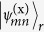 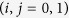 should ideally return to the vacuum in step (x). The resonator states at these various stages can be obtained by applying the displacements and waiting periods as described in the Fig. 1(a), where the time evolution is now determined by 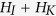, with 
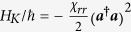
, i.e.,


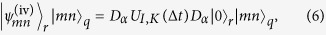



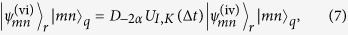



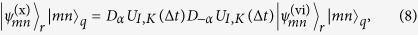


where 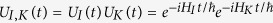 and 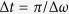. Note that the operations 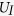 can be eliminated from (6)–(8), since 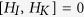 and the displacements 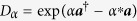 satisfy 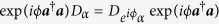. Acting on a state with fixed *m*, *n*, one therefore has


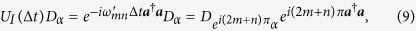


where the rotating frame frequencies are 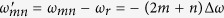. The 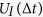 can hence be easily commuted with the displacements 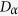 (at most changing the signs of the displacement parameters for 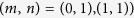. In the parameter regime that we have chosen, one further has 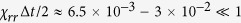, where the couplings of the two qubits to the cavity contribute to the cavity Kerr term approximately (see the Supplementary Information) as 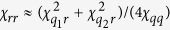. We may therefore expand 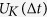 into a power series for small 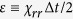, i.e.,


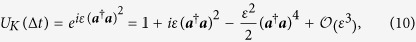


where 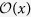 is a quantity such that 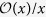 is bounded in the limit 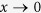. Since 
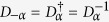
, we can then use the simple identities 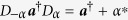 and 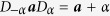. With some tedious but straightforward algebra we arrive at the estimates for the desired overlap after step (iv), i.e.,


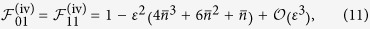


where 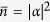 and we have used the shorthand


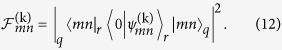


Similarly, we find the overlaps after steps (vi),


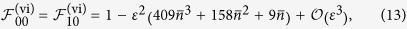


and for step (x) for all 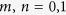:


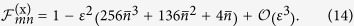


The explanation for the revival of the fidelity from step (vi) to (x) lies in the leading order Kerr effect. As discussed in [44, App. B], to linear order in *ε*, the Kerr effect does not distort the shape of the coherent states, but adds an amplitude-dependent rotation and global phase factor to each coherent state, that is


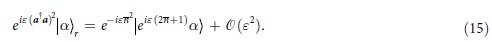


Although the phases 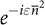 appear as relative phases between different qubit states, our protocol is designed in such a way that all qubit states acquire the same (and hence a global) phase: in steps (iii) and (ix) all components have the same amplitude 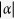, and the phases picked up by 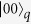 and 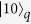 in step (v) are compensated by those acquired by 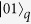 and 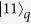 in step (vii).

The additional rotation(s) from *α* to 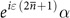, on the other hand, are only partially compensated in the protocol of Fig. 1. The coherent state components 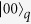 and 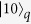 overshoot their target in steps (iii) and (v), i.e., the coherent states are rotated counter-clockwise with respect to their target on the horizontal axis. This means, the displacement 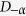 in step (viii) leaves them lagging behind, that is, rotated clockwise w.r.t. their target. This lag is partially compensated by the over-rotation in step (ix) in the sense that the mismatch in step (iii) is fully corrected, but the error incurred during step (v) remains. A similar argument applies for the components corresponding to 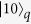 and 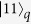, resulting in an improved fidelity in (14) as compared to (13). Nonetheless, the relatively large influence of the Kerr effect in (11)–(13) is problematic, and indeed does not justify terminating the power series after order 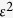. We shall hence modify our protocol in a similar fashion as discussed in^44^ to correct for the leading order Kerr effect altogether.

#### Corrected two-qubit protocol

**Figure 2:**
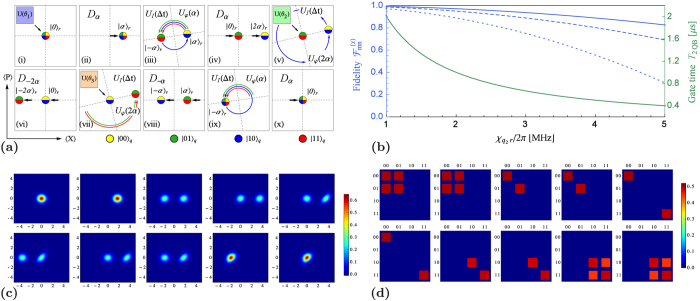
Corrected two-qubit protocol. (**a**) The ideal two-qubit protocol from Fig. 1 is modified to correct for the leading order cavity Kerr effect. After each period of free time evolution, the basis in the phase space is adjusted by an angle 
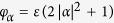
 [steps (iii) and (ix)] or 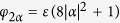 [steps (v) and (vii)], corresponding to the largest displacement in the previous step. (**b**) The gate fidelity (top three blue curves) as measured by 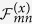 (the overlap of the final cavity states with the vacuum) and the gate time 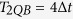 (in *μ*s, bottom green) are shown as functions of 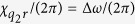 in MHz. The fidelities (not taking into account decoherence) are shown for values 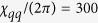 MHz, and average photon numbers 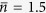 (solid, top), 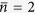 (dashed, second from the top), and 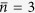 (dotted, third from the top). (**c**) A simulation (using the QuTiP library^59^) of the Wigner function of the reduced cavity state in the corrected protocol steps (i)–(x) from (**a**) is shown for 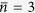, 
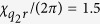
 MHz, and 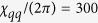 MHz [corresponding to the third curve from the top in (**b**)] when dephasing and amplitude damping for the qubits (with coherence times 
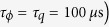
, and photon loss for the resonator 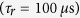 are taken into account. Note that even without loss the reduced cavity state 
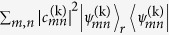
 does not feature interference fringes. In addition, the application of the conditional qubit unitaries can decrease the interference between the vacuum and excited state components in the reduced cavity state, partially restoring the rotational symmetry of the Wigner function. This can be seen, e.g., in the transition from step (vi) to (vii) in (**c**). The simulated protocol, for which the reduced qubit state amplitudes 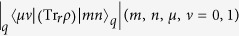 are shown in (**d**), was run for the initial qubit state 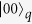, and with rotation angles 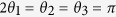. Including decoherence, the simulation with 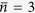, 
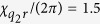
 MHz, and 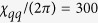 MHz yielded 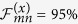 and the overlap of the final state of the qubits with the target state amounted to 93%. Further details on this, and additional simulations with other choices of parameters can be found in the Supplementary Information.

To completely compensate for the contribution of the linear order Kerr effect, the displacements after each period of free time evolution are adjusted by angles 
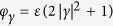
, where *γ* is the maximal displacement of the coherent state components during the time evolution. Alternatively, this may be seen as a suitable change of basis in the phase space, as illustrated in Fig. 2(a). For the evaluation of the fidelities, we hence include the transformations 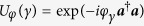, that is


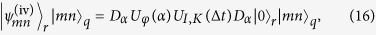



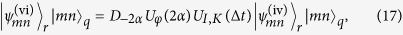



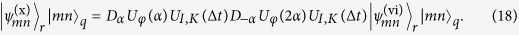


Proceeding as before, we arrive at the corrected fidelities for the overlap with the vacuum after steps (iv), (vi), and (x), given by


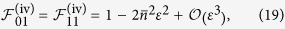



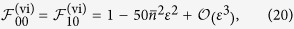



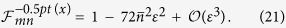


The fidelity of the protocol as quantified by 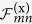 (not yet taking into account decoherence) hence also depends on the displacement via the average photon number 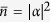. Crucially, these values have to be chosen such that the overlaps between the different coherent state components remain small in steps (v) and (vii). For values 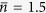 and 2 one finds 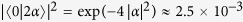 and 3.3 × 10^−4^, respectively. For 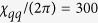 MHz and 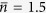, one may reach fidelities 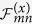 between 99% and 94% for qubit-cavity cross-Kerr coefficients ranging from 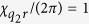 MHz to 3 MHz, see Fig. 2(b). In the Supplementary Information we further include decoherence effects — dephasing and amplitude damping of the qubits with coherence times 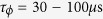 and 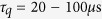, respectively, as well as photon loss in the cavity with coherence times 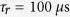 , but we consider the single-qubit operations to be perfect. Employing simulations coded in PYTHON using the QuTiP library^59^ we find that fidelities of 95% are reasonably achievable, e.g., for 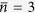, 
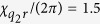
 MHz, 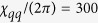 MHz and coherence times of 100 *μ*s for a range of initial states and angles 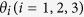, e.g., as shown in Fig. 2(c,d).

#### Other corrections

Before we finally turn to the extension to three (and more) qubits, let us remark on additional possible sources of decreases in fidelity. In a similar way, in which the cavity inherits the Kerr term from the anharmonicities of the qubits, the qubits are also coupled via the cavity, even if direct capacitive coupling can be avoided by arranging the qubits to be spatially well-separated. The corresponding term of the form 
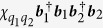
 leads to additional phases that are acquired by the components 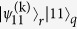 during the time evolution. This effect is at most of a size comparable to the cavity Kerr effect, which is required to be small to achieve high-fidelities. However, when the effect becomes non-negligible, it can be corrected by applying an appropriate phase gate along with 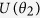 during step (v). In a regime where this becomes necessary, additional measures are further required to compensate the cavity Kerr effect beyond the linear order corrections that we have considered so far. If required, this can be achieved by a scheme, recently proposed in Ref. 60, that relies on additional ancilla qubits. In the strong dispersive regime, the frequency shift of the ancilla qubit due to different photon numbers in the cavity is used for photon-number selective phase gates that compensate the phases acquired by the different Fock state components of the coherent states. When such corrections are used to eliminate the cavity self-Kerr effect, the fidelity 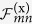 of our protocol with parameters as in Fig. 2 can reach 99%.

Another potential source of errors lies in imperfections in the circuit fabrication that may cause a small deviation *δ* from the desired ratio 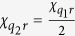 of the qubit-cavity cross-Kerr coefficients, that is, one may encounter a situation where 
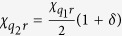
 for some small (real) 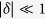. This causes an additional rotation for the coherent state components corresponding to 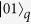 and 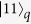 in steps (iii), (vii) and (ix), but not for the components corresponding to 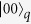 and 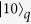. For steps (iii) and (vii) this effect can be compensated by modified displacements in steps (iv), (vi) and (viii). However, to recombine all coherent state components in step (ix), an “echo”-type operation is required. That is, step (ix) is amended in the following way: after a waiting time 
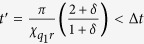
 an unconditional *π*-pulse^44^ is applied to the second qubit, which exchanges the subspaces 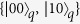 and 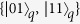. After another waiting period of duration 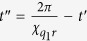 a final *π*-pulse to the second qubit restores the original qubit state. Step (x) can then be executed with an appropriately modified displacement to complete the protocol. With the possibility for these corrections in mind, we now turn to the three-qubit protocol.

### Coherent controlization for three qubits & scalability

**Figure 3:**
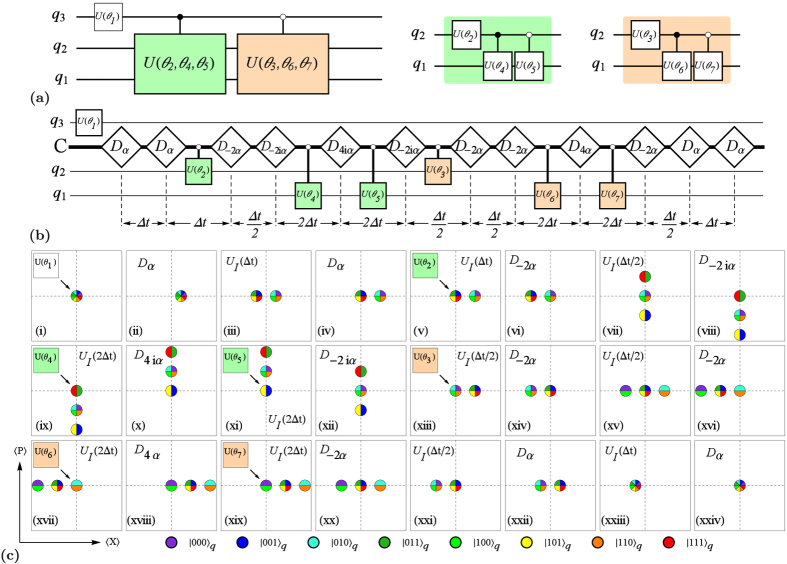
Three-qubit protocol for coherent controlization. The circuit shown in (**a**), where the filled dots “•” on the controlled operations indicate that the unitaries on the target are conditioned on the control qubit state 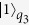, while the hollow dots “◦” denote conditioning on the control qubit state 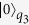, represents a three-qubit probability unitary 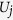. (**b**) shows an extended circuit diagram that realizes the circuit in (**a**) by adding control to the single-qubit *Y*-rotations 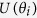. The uppermost, and the two lowest horizontal lines indicate qubits 3, 2 and 1, respectively, while the second line from the top represents the cavity mode. The white, diamond-shaped rectangles represent unconditional displacements of all cavity modes, and the control lines between the cavity and the single-qubit operations indicate operations conditioned on the resonator mode being in the ground state. The waiting time between the displacements varies between 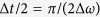 and the full period 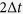. (**c**) shows the phase space representation of the coherent state components 
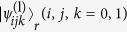
 corresponding to the qubit states 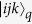 for the steps 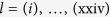 of the ideal three-qubit protocol.

Let us now discuss the extension of our protocol to 3 qubits and beyond. We will consider the ideal-three qubit protocol for the realization of a probability unitary 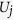 as a proof-of-principle example from which the construction of the protocol for any number of qubits may be inferred. To extend the previous protocol, first note that the cross-Kerr interaction 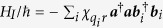 again splits the resonator frequency into spectral lines for all the qubit states. The sidebands are equally spaced by 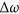 by selecting 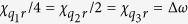. The ideal protocol, detailed in Fig. 3, then relies on the same type of operations as before, *unconditional displacements* (now with amplitudes up to 4*α*), and *waiting periods* (now of durations 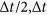, and 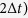, and qubit operations conditioned on the cavity being in the vacuum state.

Increasing the number of qubits hence brings about exponential scaling of several quantities of interest — as expected when exponentially increasing the dimension of the state space that we wish to explore. To separate the coherent state components corresponding to all different subspaces, larger displacements and finer graining of the rotation angles in phase space are required. That is, in addition to rotations by half-periods, for three qubits also quarter periods are necessary, and for *n*-qubits, rotations by 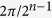 are needed. In addition, waiting periods 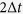 corresponding to the full period become obligatory when applying the conditional qubit rotations. Starting from an ideal *n* qubit protocol (for 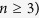, an additional qubit may be added by inserting 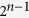 sequences of operations between each pair of conditioned unitaries on the qubit previously labelled *q*_1_ (e.g., 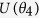 and 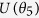 for 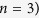. Each sequence consists of (at most) 4 displacements and two conditioned unitaries on the new qubit and the waiting periods after the 4 new displacements are of duration 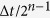, 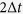, 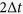 and 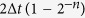, respectively. We hence find that, in an ideal *n*-qubit protocol, the number of displacements is 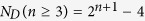, the number of qubit unitaries is 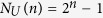, and the overall duration is 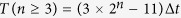. In a nonideal protocol, correction operations have to be included as well. For instance, for each conditioned qubit unitary 2 echo pulses (as discussed in the previous section) may need to be added, increasing the total number of qubit operations to 3 × 2*^n^*^−1^, but leaving the gate time unchanged. The exponential increase in dimension hence carries over to the scaling of the number of conditional qubit operations, and to the overall gate time. Since increases in gate times, displacements and qubit-cavity cross-Kerr coefficients 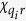 all lead to increased disturbance due to the cavity Kerr effect, its compensation using photon-number selective phase gates^60^ becomes ubiquitous, despite the possibility to correct for the linear order Kerr effect in Eq. (15) even in the presence of three individual phase space components with different displacements.

Nonetheless, it should be mentioned that such challenges for the scaling of quantum computational architectures are expected for every physical platform, and are hence not specific to our proposal. For our three-qubit protocol, simulations (using the QuTiP library^59^) that take into account partial correction of the linear Kerr effect and decoherence yield fidelities for the cavity state of 80% (64% for the qubits) for the system parameters 
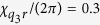
 MHz, 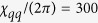 MHz and 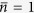, see the Supplementary Information. However, when corrections as discussed in^60^ are included, the same parameters yield fidelities of up to 95% for the cavity, and 75% for the qubits.

## Discussion

We have introduced a protocol for coherent controlization, that is, adding control to (a set of) unspecified or unknown unitaries, using superconducting qubits coupled to a microwave resonator. This task is of interest for the flexible realization of quantum computational architectures, but also of great importance for the adaptiveness of quantum-enhanced learning agents^48^. We have selected an example from the latter context, the reflective projective simulation model^15^ for artificial intelligence, where coherent controlization is already useful at the lowest level of the deliberation algorithm to construct coherent encodings of Markov chains — the probability unitaries.

We have given explicit protocols for the realization of these unitary operations using transmon qubits^44^. Within the strong dispersive regime, we exploit the coupling between the qubits and the resonator. The cavity mode here acts as a bus between the qubits, playing the role of the additional degree of freedom necessary for adding control to unknown unitaries, similar, e.g., to the vibrational modes of trapped ions^51^. We have provided a detailed discussion of the role of the cavity Kerr effect, the strongest source of disturbance, in our protocol, including corrections for the linear order effect. Based on these considerations, and bolstered by numerical simulation including photon loss, as well as amplitude- and phase-damping for the qubits (featured in the Supplementary Information), we conclude that a possible experimental realization of our protocol with two qubits may achieve high fidelities (up to 95% for the cavity and 93% for the qubits) for reasonable ranges of the system parameters. We hence consider our proposal for two qubits to be readily implementable using current superconducting technology. For three (or more) qubits, an implementation is still possible, although the significant drop in fidelity (<80% and <64% for the cavity and qubits respectively) suggests that upscaled versions of our protocol may require additional corrections of the Kerr effect^60^, which can bring the fidelities up to 95% (cavity) and 75% (qubits).

## Additional Information

**How to cite this article**: Friis, N. *et al.* Coherent controlization using superconducting qubits. *Sci. Rep.* **5**, 18036; doi: 10.1038/srep18036 (2015).

## Supplementary Material

Supplementary Information
