# Supplementary material for: Coherent controlization using superconducting qubits
Source: Sci Rep. 2015 Dec 15;5:18036. doi: 10.1038/srep18036 (PMC4678369; doi:10.1038/srep18036)
Supplement: Supplementary Information [file srep18036-s1.pdf]

Coherent controlization using superconducting qubits
Supplementary Information

Nicolai Friis,[1,*] Alexey A. Melnikov,[1,2] Gerhard Kirchmair,[2,3] and Hans J. Briegel[1]

[1] *Institute for Theoretical Physics, University of Innsbruck, Technikerstraße 21a, A-6020 Innsbruck, Austria*
[2] *Institute for Quantum Optics and Quantum Information, Austrian Academy of Sciences, Technikerstraße 21a, A-6020 Innsbruck, Austria*
[3] *Institute for Experimental Physics, University of Innsbruck, Technikerstraße 25, A-6020 Innsbruck, Austria*

(Dated: October 12, 2015)

The Supplementary Information is structured as follows. In Sec. A we provide a derivation of the Hamiltonian of the transmon qubits coupled to a resonator. The aim of this section is to give a transparent account of all approximations made in the system we consider and to give an introduction to this kind of superconducting qubits that is accessible for non-specialists. In Sec. B we then present the details of the simulations that were conducted to assess the robustness of our protocol in the presence of the Kerr effect, dephasing and amplitude damping of the qubits, and photon loss in the resonator.

A. TRANSMON QUBITS COUPLED TO A MICROWAVE RESONATOR

A.I. From Josephson junctions to transmons

A superconducting LC–circuit may be thought of as a harmonic oscillator, where the position and conjugate momentum variables are the flux $\boldsymbol{\Phi}$ through the inductor and the charge $\boldsymbol{Q}$ on the capacitor plates, respectively. Indeed, these quantities have to be treated as operators satisfying the canonical commutation relation $[\boldsymbol{\Phi}, \boldsymbol{Q}] = i\hbar$, see, e.g., Ref. [1]. In other words, the system Hamiltonian $H = \boldsymbol{Q}^2/(2C) + \boldsymbol{\Phi}^2/(2L)$ may be written as

$$H = \hbar\omega(\boldsymbol{q}^\dagger\boldsymbol{q} + 1/2) \tag{A.1}$$

in terms of the ladder operators defined by

$$\boldsymbol{Q} = -i\sqrt{\frac{\hbar}{2Z}}\left(\boldsymbol{q} - \boldsymbol{q}^\dagger\right), \tag{A.2a}$$

$$\boldsymbol{\Phi} = \sqrt{\frac{\hbar Z}{2}}\left(\boldsymbol{q} + \boldsymbol{q}^\dagger\right), \tag{A.2b}$$

with $\omega = 1/\sqrt{LC}$, $Z = \sqrt{L/C}$, and $\left[\boldsymbol{q}, \boldsymbol{q}^\dagger\right] = 1$. The introduction of a Josephson junction (a thin insulating barrier separating two pieces of superconducting material) creates a non-linearity in the system that allows identifying two energy levels as the qubit levels. The Hamiltonian is then modified to

$$H = \frac{\boldsymbol{Q}^2}{2C_\Sigma} - \frac{\Phi_0^2}{L_J}\cos\left(\frac{\boldsymbol{\Phi}}{\Phi_0}\right), \tag{A.3}$$

where the junction inductance L_J is typically expressed via the Josephson energy E_J, that is, $L_J = \Phi_0^2/E_J$, with the magnetic flux quantum $\Phi_0 = \hbar/(2e)$, and $\boldsymbol{\Phi}/\Phi_0$ is the phase difference across the junction. The original capacitance and the Josephson junction now form a superconducting island — a Cooper-pair box (CPB) — with capacitance $C_\Sigma = C + C_J$, which can be written in terms of the charging energy $E_C = e^2/(2C_\Sigma)$. The charge in the CPB depends on $\boldsymbol{n}$, the number of transferred Cooper pairs (with charge $2e$), and the effective offset charge $2en_o$, i.e., $\boldsymbol{Q}/(2e) = \boldsymbol{n} - n_o$. With the replacement $Z \to Z_J = \sqrt{L_J/C_\Sigma}$ one may define raising and lowering operators in full analogy to (A.2). To remove the strong dependence of the energy-level splitting on the (environmentally induced) offset charge, an additional shunting capacitor with large capacitance C_B can be inserted into the circuit in parallel with the Josephson junction to create the *transmon qubit* [2]. This strongly increases $C_\Sigma \to C_\Sigma = C_B + C + C_J$, such that $E_C/E_J \ll 1$. Inserting the ladder operators $\boldsymbol{q}$ and $\boldsymbol{q}^\dagger$ (with $Z \to Z_J$) into Eq. (A.3) and expanding in powers of $\sqrt{E_C/E_J}$ we arrive at

$$\begin{aligned} H = &\sqrt{2E_JE_C} - E_J - \frac{E_C}{4} + \left(\sqrt{8E_JE_C} - E_C\right)\boldsymbol{q}^\dagger\boldsymbol{q} \\ &- \frac{E_C}{3}\left(\boldsymbol{q}^\dagger\boldsymbol{q}^\dagger\boldsymbol{q}^\dagger\boldsymbol{q} + \boldsymbol{q}^\dagger\boldsymbol{q}\boldsymbol{q}\boldsymbol{q}\right) - \frac{E_C}{12}\left(\boldsymbol{q}\boldsymbol{q}\boldsymbol{q}\boldsymbol{q} + \boldsymbol{q}^\dagger\boldsymbol{q}^\dagger\boldsymbol{q}^\dagger\boldsymbol{q}^\dagger\right) \\ &- \frac{E_C}{2}\left(\boldsymbol{q}\boldsymbol{q} + \boldsymbol{q}^\dagger\boldsymbol{q}^\dagger + \boldsymbol{q}^\dagger\boldsymbol{q}^\dagger\boldsymbol{q}\boldsymbol{q}\right). \end{aligned} \tag{A.4}$$

Within the rotating wave approximation, the Hamiltonian of Eq. (A.4) can be truncated to the effective transmon qubit Hamiltonian

$$H_q = \hbar\omega_Q\,\boldsymbol{q}^\dagger\boldsymbol{q} - \frac{\hbar\beta}{2}\boldsymbol{q}^\dagger\boldsymbol{q}^\dagger\boldsymbol{q}\boldsymbol{q}, \tag{A.5}$$

where $\omega_Q \equiv \left(\sqrt{8E_JE_C} - E_C\right)/\hbar$ is the transmon qubit frequency and $\beta \equiv E_C/\hbar$ is the anharmonicity.

* nicolai.friis@uibk.ac.at

A.II. Transmons coupled to a resonator

Next, we are interested in describing a transmon qubit that is capacitively coupled to a microwave resonator. Within the rotating wave approximation the joint system is described by the Hamiltonian

$$H_{qr}/\hbar = \omega_R \boldsymbol{c}^\dagger \boldsymbol{c} + \omega_Q \boldsymbol{q}^\dagger \boldsymbol{q} + g\left(\boldsymbol{c}^\dagger \boldsymbol{q} + \boldsymbol{c}\boldsymbol{q}^\dagger\right) - \frac{\beta}{2}\boldsymbol{q}^\dagger\boldsymbol{q}^\dagger\boldsymbol{q}\boldsymbol{q}\,, \tag{A.6}$$

where g is the qubit-cavity coupling, ω_R and ω_Q are the frequencies of the isolated qubit and cavity, respectively, and the operators $\boldsymbol{c}$ and $\boldsymbol{c}^\dagger$ are the resonator ladder operators satisfying $[\boldsymbol{c}\,,\boldsymbol{c}^\dagger] = 1$. The terms in H_{qr} that are quadratic in the mode operators can be diagonalized by a Bogoliubov transformation. That is, one introduces the dressed operators $\boldsymbol{a}$ and $\boldsymbol{b}$, such that

$$\begin{aligned} \boldsymbol{c} &= \cos\theta\ \boldsymbol{a} + \sin\theta\ \boldsymbol{b}\,, \\ \boldsymbol{q} &= -\sin\theta\ \boldsymbol{a} + \cos\theta\ \boldsymbol{b}\,, \end{aligned} \tag{A.7}$$

with $\tan 2\theta = 2g/\Delta$, where $\Delta \equiv \omega_Q - \omega_R$ is the qubit-cavity detuning. The excitations of the dressed modes can no longer be uniquely attributed to just the resonator or just the qubit. However, in the strong dispersive regime, where $g/\Delta \ll 1$, the mixing angle is small, $\theta \approx g/\Delta$ and excitations created by $\boldsymbol{c}^\dagger$ ($\boldsymbol{q}^\dagger$) are associated "mostly" with the cavity (qubit). The Hamiltonian transforms to

$$\begin{aligned} H_{qr}/\hbar = {}& \tilde{\omega}_r \boldsymbol{a}^\dagger\boldsymbol{a} + \tilde{\omega}_q \boldsymbol{b}^\dagger\boldsymbol{b} - 2\beta\sin^2\theta\cos^2\theta\ \boldsymbol{a}^\dagger\boldsymbol{a}\boldsymbol{b}^\dagger\boldsymbol{b} \\ & - \frac{\beta}{2}\cos^4\theta\ \boldsymbol{b}^\dagger\boldsymbol{b}^\dagger\boldsymbol{b}\boldsymbol{b} - \frac{\beta}{2}\sin^4\theta\boldsymbol{a}^\dagger\boldsymbol{a}^\dagger\boldsymbol{a}\boldsymbol{a} \\ & + \beta\sin\theta\cos\theta\ \Big[\sin^2\theta\big(\boldsymbol{a}^\dagger\boldsymbol{a}^\dagger\boldsymbol{a}\boldsymbol{b} + \boldsymbol{a}^\dagger\boldsymbol{a}\boldsymbol{a}\boldsymbol{b}^\dagger\big) \\ & + \cos^2\theta\ \big(\boldsymbol{a}^\dagger\boldsymbol{b}^\dagger\boldsymbol{b}\boldsymbol{b} + \boldsymbol{a}\boldsymbol{b}^\dagger\boldsymbol{b}^\dagger\boldsymbol{b}\big) \\ & + \sin\theta\cos\theta\ \big(\boldsymbol{a}^\dagger\boldsymbol{a}^\dagger\boldsymbol{b}\boldsymbol{b} + \boldsymbol{a}\boldsymbol{a}\boldsymbol{b}^\dagger\boldsymbol{b}^\dagger\big)\Big]\,, \end{aligned} \tag{A.8}$$

where the dressed mode frequencies are given by

$$\tilde{\omega}_r = \omega_R\cos^2\theta + \omega_Q\sin^2\theta - g\sin(2\theta)\,, \tag{A.9a}$$

$$\tilde{\omega}_q = \omega_R\sin^2\theta + \omega_Q\cos^2\theta + g\sin(2\theta)\,. \tag{A.9b}$$

The terms in the square bracket can be seen to oscillate rapidly, and we may therefore remove these terms in another rotating wave approximation. With the notation $\chi_{qr} \equiv (\beta/2)\sin^2(2\theta)$, $\chi_{qq} \equiv \beta\cos^4\theta$, and

$$\chi_{rr} \equiv \frac{\chi_{qr}^2}{4\chi_{qq}} = \beta\sin^4\theta\,, \tag{A.10}$$

we then arrive at the Hamiltonian of a single transmon qubit coupled to a resonator in the dispersive limit

$$\begin{aligned} H_{qr}/\hbar = {}& \tilde{\omega}_r\boldsymbol{a}^\dagger\boldsymbol{a} + \tilde{\omega}_q\boldsymbol{b}^\dagger\boldsymbol{b} - \frac{\chi_{qq}}{2}\boldsymbol{b}^\dagger\boldsymbol{b}^\dagger\boldsymbol{b}\boldsymbol{b} - \chi_{qr}\boldsymbol{a}^\dagger\boldsymbol{a}\boldsymbol{b}^\dagger\boldsymbol{b} \\ & - \frac{\chi_{rr}}{2}\boldsymbol{a}^\dagger\boldsymbol{a}^\dagger\boldsymbol{a}\boldsymbol{a} \\ = {}& \omega_r\boldsymbol{a}^\dagger\boldsymbol{a} + \omega_q\boldsymbol{b}^\dagger\boldsymbol{b} - \frac{\chi_{qq}}{2}\left(\boldsymbol{b}^\dagger\boldsymbol{b}\right)^2 - \chi_{qr}\boldsymbol{a}^\dagger\boldsymbol{a}\boldsymbol{b}^\dagger\boldsymbol{b} \\ & - \frac{\chi_{rr}}{2}\left(\boldsymbol{a}^\dagger\boldsymbol{a}\right)^2\,, \end{aligned} \tag{A.11}$$

where $\omega_r = \tilde{\omega}_r + \chi_{rr}/2$ and $\omega_q = \tilde{\omega}_q + \chi_{qq}/2$. The dressed qubit anharmonicity χ_{qq}, the qubit-cavity cross-Kerr coefficient χ_{qr}, and the cavity self-Kerr coefficient χ_{rr} can be expressed directly via the coupling strength g and the detuning Δ as

$$\chi_{qq} = \frac{\beta}{4}\left(1 + \frac{|\Delta|}{\sqrt{\Delta^2 + 4g^2}}\right)^2\,, \tag{A.12a}$$

$$\chi_{qr} = \frac{\beta}{2}\frac{4g^2}{\Delta^2 + 4g^2}\,, \tag{A.12b}$$

$$\chi_{rr} = \frac{\beta}{4}\left(1 - \frac{|\Delta|}{\sqrt{\Delta^2 + 4g^2}}\right)^2\,. \tag{A.12c}$$

A.III. Two transmons coupled to a cavity

When two transmon qubits are coupled to the cavity, we may write an analogue expression to Eq. (A.6). That is, in the rotating wave approximation we have the Hamiltonian

$$\begin{aligned} H_{qqr}/\hbar = {}& \omega_R\,\boldsymbol{c}^\dagger\boldsymbol{c} + \omega_{Q_1}\,\boldsymbol{q}_1^\dagger\boldsymbol{q}_1 + \omega_{Q_2}\,\boldsymbol{q}_2^\dagger\boldsymbol{q}_2 \\ & + g_1\left(\boldsymbol{c}^\dagger\boldsymbol{q}_1 + \boldsymbol{c}\boldsymbol{q}_1^\dagger\right) + g_2\left(\boldsymbol{c}^\dagger\boldsymbol{q}_2 + \boldsymbol{c}\boldsymbol{q}_2^\dagger\right) \\ & - \frac{\beta_1}{2}\boldsymbol{q}_1^\dagger\boldsymbol{q}_1^\dagger\boldsymbol{q}_1\boldsymbol{q}_1 - \frac{\beta_2}{2}\boldsymbol{q}_2^\dagger\boldsymbol{q}_2^\dagger\boldsymbol{q}_2\boldsymbol{q}_2\,, \end{aligned} \tag{A.13}$$

where we have neglected any direct coupling of the qubits to each other. In the two-qubit case (and beyond), an analytical diagonalization of the harmonic part (terms quadratic in the mode operators) becomes infeasible. However, using numerical methods and following similar arguments as presented in the previous Sec. A.II one arrives at the effective Hamiltonian

$$\begin{aligned} H_{qqr}/\hbar = {}& \omega_r\boldsymbol{a}^\dagger\boldsymbol{a} + \sum_i \omega_{q_i}\boldsymbol{b}_i^\dagger\boldsymbol{b}_i - \sum_i \chi_{q_i r}\,\boldsymbol{a}^\dagger\boldsymbol{a}\,\boldsymbol{b}_i^\dagger\boldsymbol{b}_i \\ & - \sum_i \frac{\chi_{q_i q_i}}{2}\left(\boldsymbol{b}_i^\dagger\boldsymbol{b}_i\right)^2 - \frac{\chi_{rr}}{2}\left(\boldsymbol{a}^\dagger\boldsymbol{a}\right)^2\,. \end{aligned} \tag{A.14}$$

Assuming that the transmons can be fabricated such that $\chi_{q_i q_i} \equiv \chi_{qq}$ for all qubits, we have numerically checked that the cavity self-Kerr coefficient χ_{rr} can be approximated as

$$\chi_{rr} \approx \frac{\chi_{q_1 r}^2 + \chi_{q_2 r}^2}{4\chi_{qq}}\,. \tag{A.15}$$

B. SIMULATIONS OF COHERENT CONTROLIZATION USING TRANSMON QUBITS

In this section we present the simulations of our protocol for coherent controlization with two and three qubits. In the simulations, which were coded in PYTHON using the QuTiP library [3], we discuss the influence of the cavity Kerr effect, including the (partial) correction of the linear Kerr effect by way of reference frame adjustments, and the possibility for correcting it entirely using photon-number selective gates [4]. In addition, all simulations assume the presence of amplitude and phase damping for the qubits, and photon loss in the resonator. The unconditional displacements can safely be assumed to be perfect. We further assume the single-qubit operations to be perfect, given that the pulses are slow enough to address only the zero-photon subspace, but short enough to fit within the Δt time intervals of our protocol.

B.I. Setup for simulations

For the simulation we truncate the Hilbert space of the resonator to be spanned by the Fock states $|n\rangle_r$ of photon numbers $n = 0, 1, \ldots, 100$. This is a good approximation since the maximal average photon number of the coherent states in our protocol is $\bar{n}_{max} = |2\alpha|^2 = 14$ ($\bar{n}_{max} = |4\alpha|^2 = 32$) photons for 2 (3) qubits and the overlap with photon numbers larger than 100 is hence negligible. For the Hilbert space of the transmon qubits we will each only consider the lowest two eigenstates, i.e., the qubit levels. The effective Hamiltonian in the frame rotating with ω_r and ω_q for the resonator and qubit Hilbert spaces, respectively, is given by

$$\frac{H_{I,K}}{\hbar} = \frac{H_I + H_K}{\hbar} = -\sum_i \chi_{q_i r}\, \boldsymbol{a}^\dagger \boldsymbol{a}\, \boldsymbol{b}_i^\dagger \boldsymbol{b}_i - \frac{\chi_{rr}}{2}\left(\boldsymbol{a}^\dagger \boldsymbol{a}\right)^2 = -\sum_i \chi_{q_i r}\, \boldsymbol{a}^\dagger \boldsymbol{a}\, \sigma_i^+ \sigma_i^- - \frac{\chi_{rr}}{2}\left(\boldsymbol{a}^\dagger \boldsymbol{a}\right)^2, \tag{B.16}$$

where $\sigma_j^\pm = \sigma_j^x \pm i\sigma_j^y$ are the raising/lowering operators of the j-th qubit. In addition to the free time evolution we will include corrections for the linear order of the Kerr effect by including rotations $U_\varphi(\gamma) = \exp(-i\varphi_\gamma \boldsymbol{a}^\dagger\boldsymbol{a})$ after each period of time evolution, where γ is the maximal displacement of the different coherent state components in the preceding step of the protocol. The conditional single-qubit operations will be represented by

$$|0\rangle\langle 0|_r \otimes U_q(\theta_i) + \left(\mathbb{1}_r - |0\rangle\langle 0|_r\right) \otimes \mathbb{1}_q, \tag{B.17}$$

where the single-qubit Y-rotations on the second (and third) of the two (three) qubits are realized by a time-dependent drive $U(\theta_j) = \exp(-i\frac{\theta_j}{2}\sigma^y \frac{t}{T})$. The durations T of these drives are taken to be the durations of the waiting periods in the corresponding protocol steps ($T = \Delta t$ for two qubits). On top of the unitary time evolution, unconditional displacements, and conditional qubit operations, all of which will be assumed to be perfect, we will consider decoherence in the system. In particular, we assume that the dynamics of the overall state ρ of the joint cavity-qubit system during the waiting periods of the protocol is governed by the master equation

$$\frac{d\rho}{dt} = -\frac{i}{\hbar}\left[H_{I,K}, \rho\right] + \frac{1}{2\tau_r}\left(2\boldsymbol{a}\rho\boldsymbol{a}^\dagger - \boldsymbol{a}^\dagger\boldsymbol{a}\rho - \rho\boldsymbol{a}^\dagger\boldsymbol{a}\right) + \frac{1}{2\tau_q}\sum_i\left(2\boldsymbol{\sigma}_i^-\rho\boldsymbol{\sigma}_i^+ - \boldsymbol{\sigma}_i^+\boldsymbol{\sigma}_i^-\rho - \rho\boldsymbol{\sigma}_i^+\boldsymbol{\sigma}_i^-\right) + \left(\frac{1}{2\tau_\phi} - \frac{1}{4\tau_q}\right)\sum_i\left(\boldsymbol{\sigma}_i^z\rho\boldsymbol{\sigma}_i^z - \rho\right). \tag{B.18}$$

As the output of interest of the simulations we consider the fidelities $\mathcal{F}_r$ for the resonator, i.e., the squared overlap with the vacuum at the final step of the protocol, given by $\mathcal{F}_r = {}_r\langle 0|\left(\mathrm{Tr}_q\rho\right)|0\rangle_r$, and $\mathcal{F}_q$ for the qubits, i.e., the squared overlap with the target state $|\psi\rangle_q$, given by $\mathcal{F}_q = {}_q\langle\psi|\left(\mathrm{Tr}_r\rho\right)|\psi\rangle_q$. We evaluate these two fidelities separately because the success of the protocol is ultimately only determined by achieving a large overlap of the reduced qubits state with the desired target state, irrespective of the final cavity state. On the other hand, the cavity fidelity gives a more detailed overview of the errors incurred by the Kerr effect in the resonator. Therefore, both $\mathcal{F}_r$ and $\mathcal{F}_q$ are quantities of interest for our protocol. In addition to the final fidelities, we include plots of the Wigner function $W(x, y)$, where

$$W(x,y) = \frac{1}{2\pi\hbar}\int_{-\infty}^{\infty} dy\, e^{ipy/\hbar}\, {}_r\langle x - \tfrac{y}{2}|\left(\mathrm{Tr}_q\rho\right)|x + \tfrac{y}{2}\rangle_r, \tag{B.19}$$

of the reduced resonator state $\rho_r = \mathrm{Tr}_q\rho$, where $|x\rangle$ are the eigenstates of the quadrature operator $\boldsymbol{\Phi}$ with eigenvalue x, and plots of the absolute values of the density matrix elements $|{}_q\langle\mu\nu|\,\rho_q\,|mn\rangle_q|$ of the reduced qubit state $\rho_q = \mathrm{Tr}_r\rho$ throughout the steps of our protocol.

B.II. Simulations for two qubits

For two qubits, the qubit-cavity cross-Kerr coefficients $\chi_{qr} \equiv \chi_{q_1 r} = 2\chi_{q_2 r}$ are chosen in a range from $1.5 \times 2\pi$ MHz to $3 \times 2\pi$ MHz. According to the approximation in Eq. (A.15), we further set $\chi_{rr} = 5\chi_{qr}^2/(4\chi_{qq})$, where χ_{qq} is fixed to $300 \times 2\pi$ MHz. The coherence time for the resonator is set to $\tau_r = 100$ μs throughout. For the simulation results presented in Fig. B.1, we hence vary the qubit-cavity coupling, the displacements in the protocol, the decoherence times, the initial qubit state $\rho_q = |\xi\rangle\langle\xi|_q$ and the angles θ_j ($j = 1, 2, 3$) for the single-qubit operations.

In addition to the results shown in Fig. B.1, we have simulated the influence of decoherence on the protocol

when assuming that the cavity Kerr effect has been fully corrected using the methods of [4]. In that case, using the same parameters as for the simulation shown in Fig. 2 (c,d) of the main text, but setting $\chi_{rr} \equiv 0$, we obtain $\mathcal{F}_r = 99\%$ and $\mathcal{F}_q = 96\%$.

Finally, note that the parameters $\chi_{q_i r}$, $\chi_{q_i q_i}$ and χ_{rr} are derived from the quantities E_{C_i}, E_{J_i}, ω_R, and the cavity-qubit couplings g_i for $i = 1, 2$. Achieving the exact required ratio $\chi_{q_1 r} = 2\chi_{q_2 r}$ hence relies on the fine tuning of these quantities. For instance, to approximately achieve the parameters of the simulation in Fig. B.1 (c), one may set $E_{J_1} = 27.0$ GHz, $E_{J_2} = 20.1$ GHz, $E_{C_1} = E_{C_2} = 0.3$ GHz, $g_1/(2\pi) = 101$ MHz, $g_2/(2\pi) = 127$ MHz, and $\omega_R/(2\pi) = 9.16$ GHz to obtain $\chi_{q_2 r}/(2\pi) = 1.499$ MHz, $\chi_{q_1 r}/(2\pi) = 2.982$ MHz $= 1.989\ \chi_{q_2 r}$, $\chi_{q_1 q_1}/(2\pi) = 296.998$ MHz, $\chi_{q_2 q_2}/(2\pi) = 298.46$ MHz, and $\chi_{rr}/(2\pi) = 9.367$ kHz (as compared to $\sum_i \chi_{q_i r}^2/(4\chi_{q_i q_i}) = 9.34$ kHz). For the simulations, we assume that such deviations are corrected by an echo-type operation as explained in Sec. III D of the main text. Finally, note that all parameters used in the simulations are compatible with the dispersive approximation.

B.III. Simulations for three qubits

For three qubits, the qubit-cavity cross-Kerr coefficients $\chi_{qr} \equiv \chi_{q_1 r} = 2\chi_{q_2 r} = 4\chi_{q_3 r}$ are chosen in a range from $0.2 \times 2\pi$ MHz to $0.4 \times 2\pi$ MHz. According to the approximation in Eq. (A.15), we further set $\chi_{rr} = 21\chi_{qr}^2/(4\chi_{qq})$, where χ_{qq} is fixed to $300 \times 2\pi$ MHz. The coherence times for the resonator and the qubit (dephasing and amplitude damping) are set to 100 μs. For the simulation results presented in Table B.1, we hence vary the qubit-cavity coupling, the displacements in the protocol, the initial qubit state $\rho_q = |\xi\rangle\langle\xi|_q$ and the angles θ_j $(j = 1, 2, \ldots, 7)$ for the single-qubit operations. A selection of these simulations Figs. B.3 and B.4 further illustrate the reduced states of the resonator and the qubits throughout the protocol.

#	χ_{qr} [MHz]	α	$\|\xi\rangle_q$	θ_1	θ_2	θ_3	θ_4	θ_5	θ_6	θ_7	$\mathcal{F}_r$	$\mathcal{F}_q$
s_1	$0.3 \times 2\pi$	1	$\|\xi_u\rangle$	0	0	0	0	0	0	0	0.671	0.296
s_2	$0.3 \times 2\pi$	1	$\|010\rangle$	$\frac{\pi}{2}$	π	0	0	π	0	$\frac{\pi}{2}$	0.505	0.449
s_3	$0.4 \times 2\pi$	1	$\|000\rangle$	0	0	0	0	0	0	0	0.832	1.000
s_4	$0.3 \times 2\pi$	1	$\|000\rangle$	$\frac{\pi}{2}$	0	$\frac{\pi}{6}$	0	π	0	$\frac{\pi}{2}$	0.699	0.481
s_5	$0.4 \times 2\pi$	1	$\|000\rangle$	$\frac{\pi}{2}$	0	$\frac{\pi}{6}$	0	π	0	$\frac{\pi}{2}$	0.578	0.457
s_6	$0.2 \times 2\pi$	1	$\|000\rangle$	$\frac{\pi}{2}$	0	$\frac{\pi}{6}$	0	π	0	$\frac{\pi}{2}$	0.747	0.444
s_7	$0.35 \times 2\pi$	1	$\|000\rangle$	$\frac{\pi}{2}$	0	$\frac{\pi}{6}$	0	π	0	$\frac{\pi}{2}$	0.646	0.475
s_8	$0.3 \times 2\pi$	1	$\|000\rangle$	π	0	0	0	0	0	π	0.697	0.797
s_9	$0.3 \times 2\pi$	$\sqrt{\frac{1}{2}}$	$\|000\rangle$	π	0	0	0	0	0	π	0.674	0.729
s_{10}	$0.3 \times 2\pi$	$\sqrt{\frac{3}{2}}$	$\|000\rangle$	π	0	0	0	0	0	π	0.329	0.796
s_{11}	$0.3 \times 2\pi$	1	$\|000\rangle$	$\frac{\pi}{12}$	0	$\frac{\pi}{3}$	0	0	$\frac{\pi}{2}$	π	0.804	0.639
s_{12}	$0.3 \times 2\pi$	1	$\|000\rangle$	$\frac{\pi}{12}$	0	$\frac{\pi}{3}$	0	0	$\frac{\pi}{2}$	π	0.953	0.748

TABLE B.1. **Three-qubit simulations**: The table shows the results of the numerical simulations for three qubits for various system parameters, rotation angles θ_j, and initial states, where $|\xi_u\rangle$ is the uniform superposition overall three qubit computational basis states. The last simulation, s_{12} was executed for the same parameters as s_{11}, but assuming that the cavity Kerr effect has been corrected (here: $\chi_{rr} \equiv 0$) using the photon-number selective phase gates [4].

[1] Devoret, M. H., Wallraff, A. & Martinis, J. M. Superconducting qubits: a short review. *arXiv*, cond-mat/0411174 (2004).

[2] Koch, J. *et al.* Charge-insensitive qubit design derived from the Cooper pair box. *Phys. Rev. A* **76,** 042319 (2007). *arXiv*, cond-mat/0703002.

[3] Johansson, J., Nation, P. D. & Nori, F. QuTiP 2: A Python framework for the dynamics of open quantum systems. *Comp. Phys. Comm.* **184,** 1234–1240 (2013). *arXiv*, 1211.6518 [quant-ph].

[4] Heeres, R. W. *et al.* Cavity state manipulation using photon-number selective phase gates. *Phys. Rev. Lett.* **115,** 137002 (2015). *arXiv*, 1503.01496 [quant-ph]

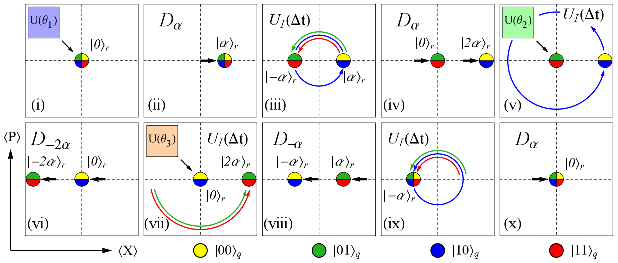

(a)

#	χ_{qr} [MHz]	α	τ_q [μs]	τ_ϕ [μs]	$\lvert\xi\rangle_q$	θ_1	θ_2	θ_3	$\mathcal{F}_r$	$\mathcal{F}_q$
**(c)**	$3\times 2\pi$	$\sqrt{2}$	20	30	$\lvert 00\rangle$	$\frac{\pi}{2}$	π	π	0.907	0.916
**(d)**	$1.5\times 2\pi$	$\sqrt{3}$	100	100	$\lvert 00\rangle$	$\frac{\pi}{2}$	$\frac{\pi}{6}$	$\frac{\pi}{3}$	0.938	0.953
**(e)**	$1.5\times 2\pi$	$\sqrt{3}$	100	100	$\lvert 00\rangle$	0	0	0	0.943	1.000
**(f)**	$1.5\times 2\pi$	$\sqrt{3}$	100	100	$\lvert 00\rangle$	$\frac{\pi}{12}$	$\frac{\pi}{4}$	π	0.955	0.953
**(g)**	$1.5\times 2\pi$	$\sqrt{3}$	100	100	$\lvert 10\rangle$	$\frac{\pi}{4}$	π	$\frac{\pi}{6}$	0.943	0.959

(b)

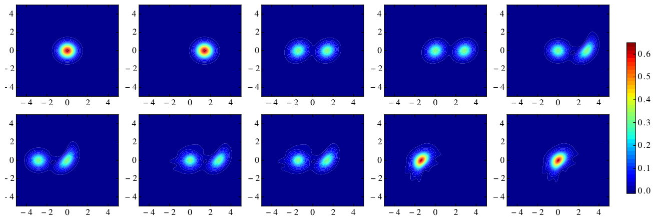

(c.1)

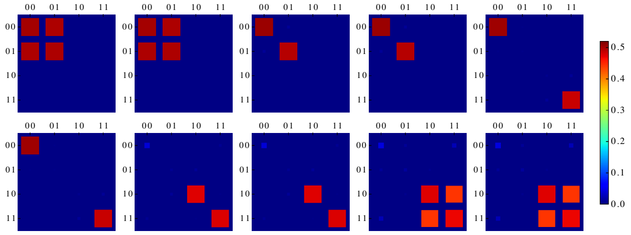

(c.2)

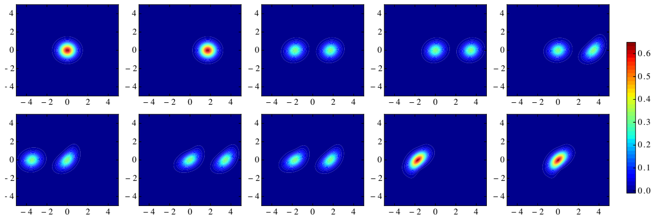

(d.1)

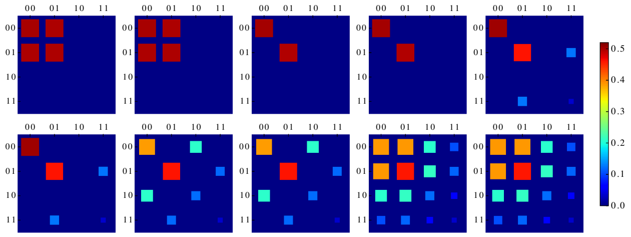

(d.2)

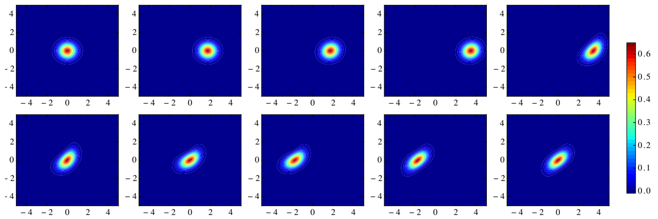

(e.1)

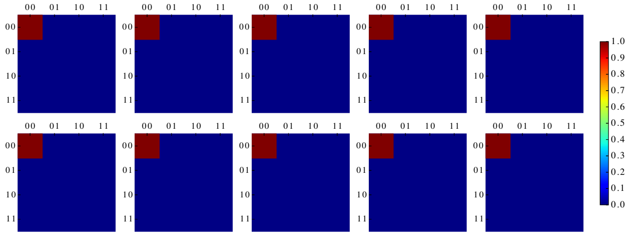

(e.2)

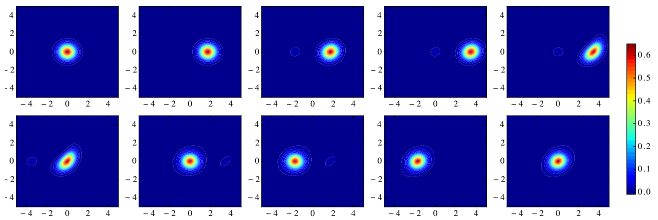

(f.1)

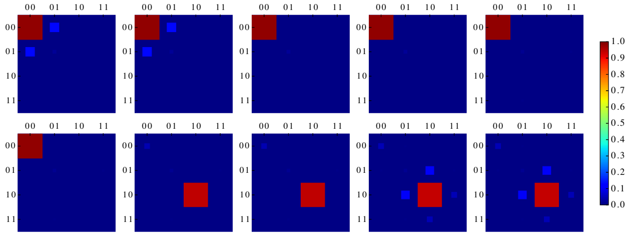

(f.2)

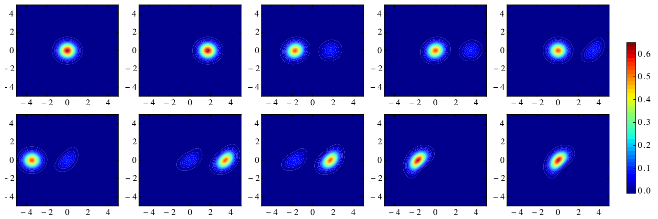

(g.1)

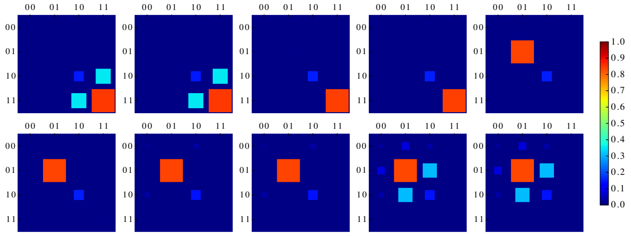

(g.2)

FIG. B.1. **Two-qubit simulations**: **(a)** Ideal protocol. **(b)** Table of simulation results. **(c)-(g)** show the reduced resonator state Wigner function on the left-hand side, and the absolute values of the density matrix elements $\lvert{}_q\langle\mu\nu\rvert\,\rho_q\,\lvert mn\rangle_q\rvert$ of the reduced qubit states on the right-hand side.

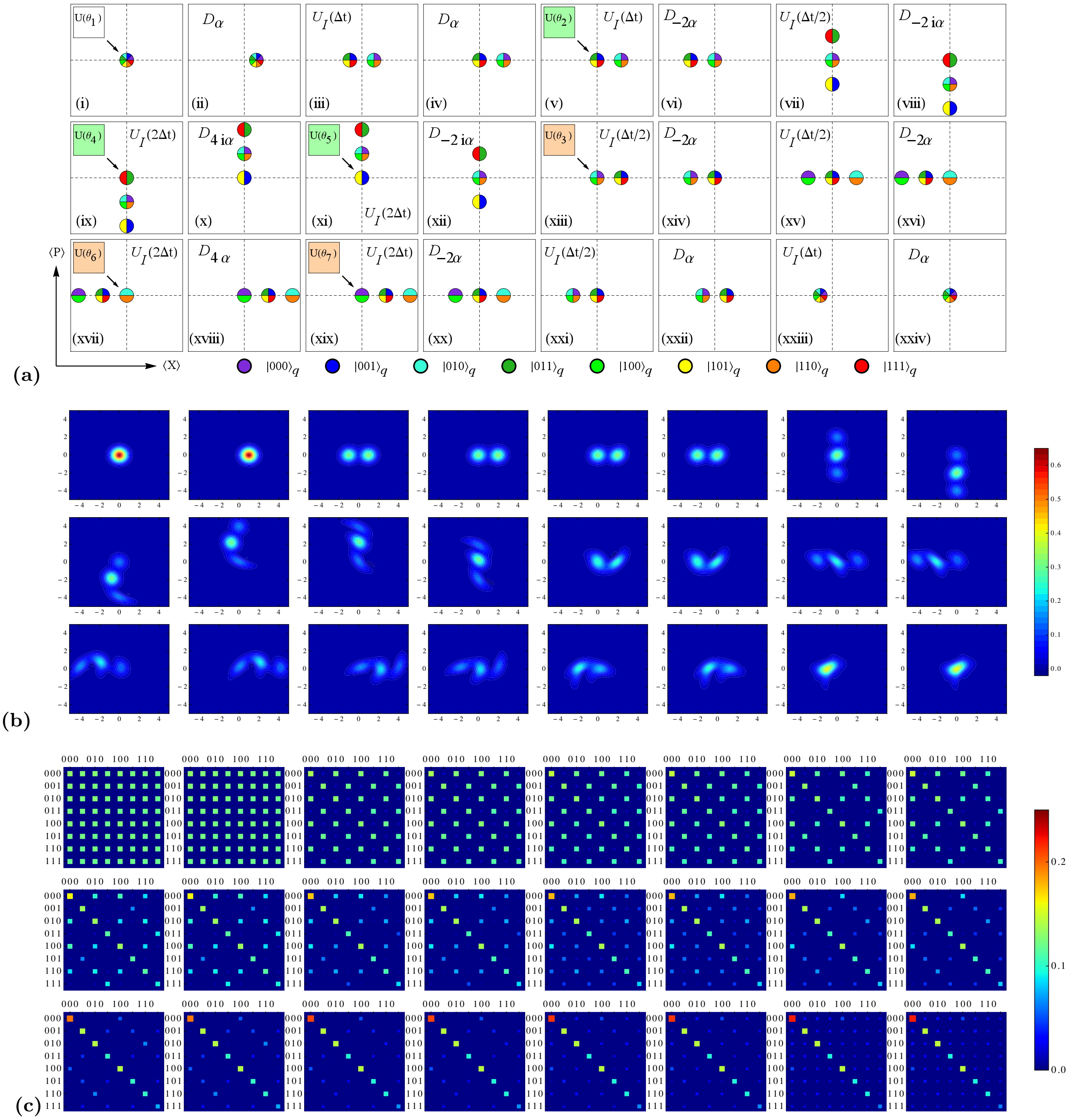

FIG. B.2. **Three-qubit simulation** s_1: **(a)** Ideal protocol. **(b)** The Wigner function of the reduced resonator state of the three-qubit protocol is shown for the parameters of the simulation specified in s_1 of Table B.1. **(c)** shows the corresponding plots of the absolute values of the density matrix elements $|_q\langle\mu\nu\lambda|\,\rho_q\,|mnl\rangle_q|$ $(\mu,\nu,\lambda,m,n,l=0,1)$ of the reduced qubit states.

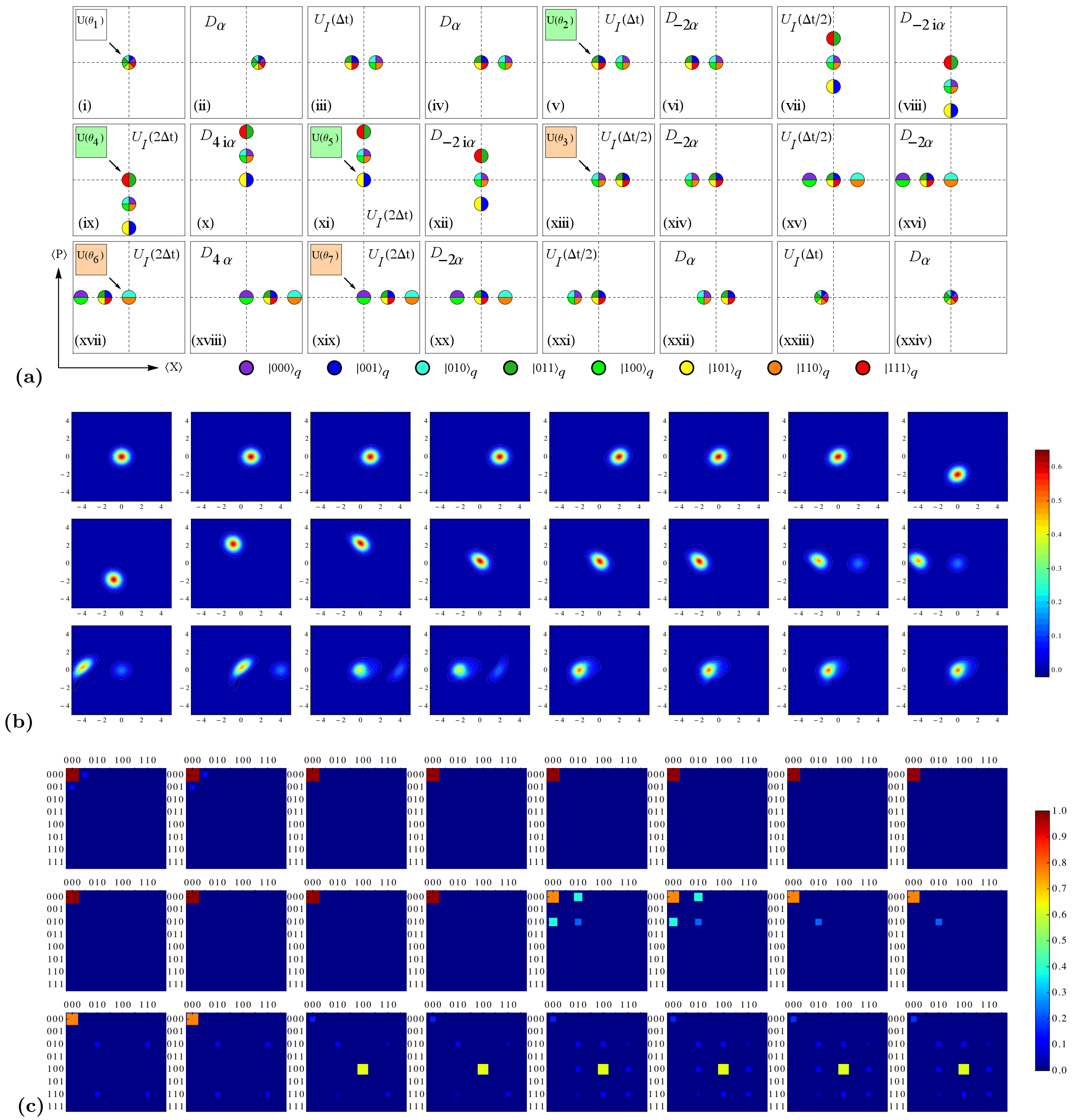

FIG. B.3. **Three-qubit simulation** s_{11}: **(a)** Ideal protocol. **(b)** The Wigner function of the reduced resonator state of the three-qubit protocol is shown for the parameters of the simulation specified in s_{11} of Table B.1. **(c)** shows the corresponding plots of the absolute values of the density matrix elements $|_q\langle\mu\nu\lambda|\,\rho_q\,|mnl\rangle_q|$ $(\mu,\nu,\lambda,m,n,l=0,1)$ of the reduced qubit states.

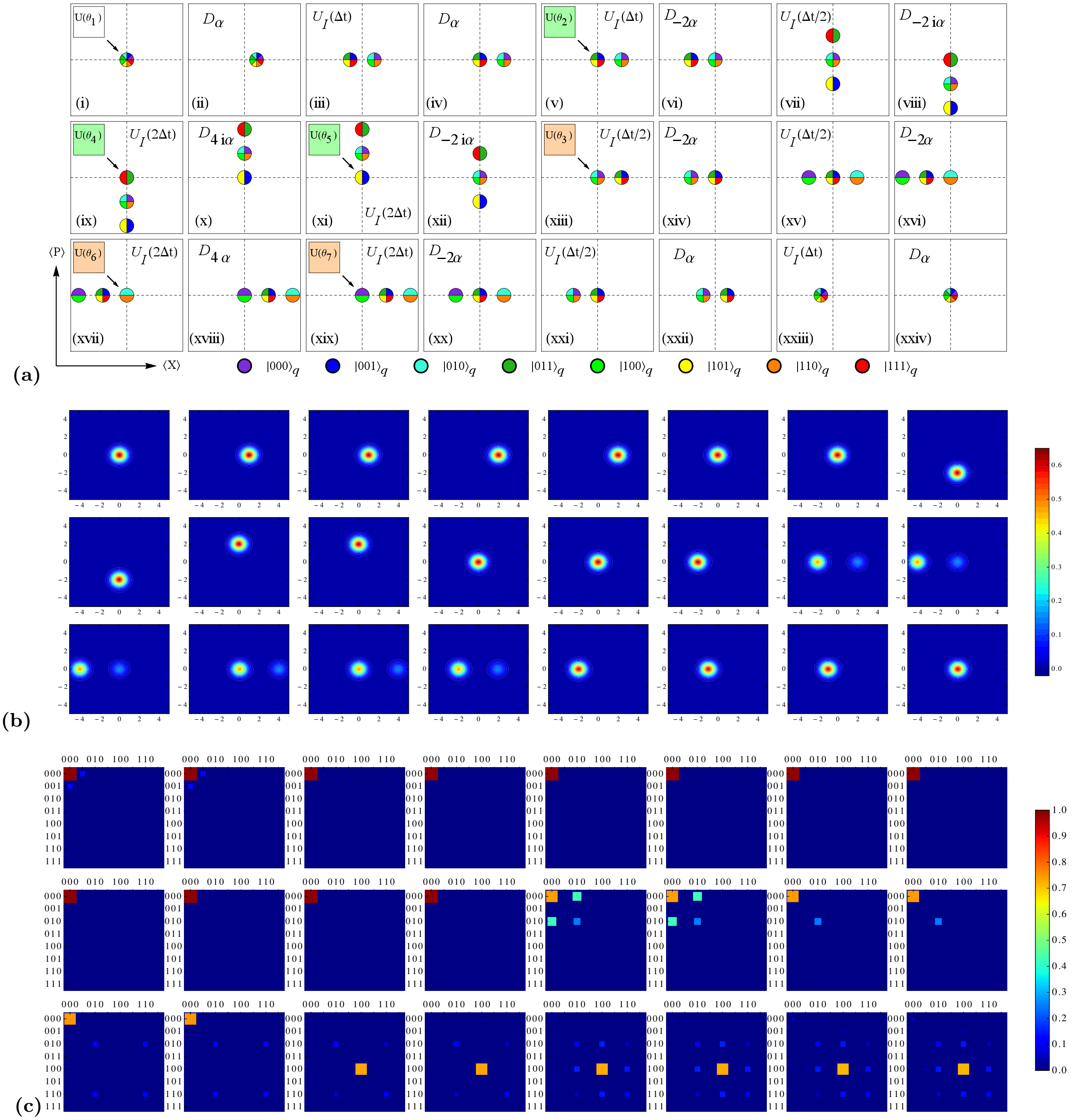

FIG. B.4. **Three-qubit simulation** s_{12}: **(a)** Ideal protocol. **(b)** The Wigner function of the reduced resonator state of the three-qubit protocol is shown for the parameters of the simulation specified in s_{12} of Table B.1, where the cavity Kerr effect is assumed to be fully corrected. **(c)** shows the corresponding plots of the absolute values of the density matrix elements $|_q\langle\mu\nu\lambda|\,\rho_q\,|mnl\rangle_q|$ $(\mu,\nu,\lambda,m,n,l=0,1)$ of the reduced qubit states.
